# Zinc Deficiency among Lactating Mothers from a Peri-Urban Community of the Ecuadorian Andean Region: An Initial Approach to the Need of Zinc Supplementation

**DOI:** 10.3390/nu10070869

**Published:** 2018-07-05

**Authors:** Camila Narváez-Caicedo, Gabriela Moreano, Bernardo A. Sandoval, Miguel Á. Jara-Palacios

**Affiliations:** 1 Escuela de Medicina, Facultad de Ciencias Médicas de la Salud y de la Vida, Universidad Internacional del Ecuador, 170113 Quito, Ecuador; camilanarvaezca@gmail.com (C.N.-C.); gaby.moreano94@gmail.com (G.M.); bsandoval@uide.edu.ec (B.A.S.); 2 Servicio de Cirugía, Hospital Metropolitano, 170521 Quito, Ecuador

**Keywords:** zinc deficiency, plasma zinc, lactating women, zinc supplementation, Quito, Ecuador, Andean region

## Abstract

Zinc is an important mineral for biological and physiological processes. Zinc deficiency (ZD) is one of the most common micronutrient deficiencies worldwide and a crucial determinant of pregnancy outcomes and childhood development. Zinc levels and the zinc supplementation rate among lactating women have not been assessed neither in Ecuador nor in the Andean region. We conducted a pilot study including 64 mothers of infants between eight days to seven months old from a primary care center located in Conocoto, a peri-urban community of Quito, Ecuador. The mothers were interviewed and a fasting blood sample was taken to determine plasma zinc levels. The prevalence of ZD was calculated and compared with the prevalence of ZD among Ecuadorian non-pregnant non-lactating women, and the sample was analysed considering zinc supplementation during pregnancy. The prevalence of ZD among the participants was 81.3% (95% CI: 71.7–90.9), higher than the reported among non-pregnant non-lactating women (G^2^ = 18.2; *p* < 0.05). Zinc supplementation rate was 31.2%. No significant differences were found comparing the groups considering zinc supplementation. The insights obtained from this study encourage extending studies to document zinc levels and its interactions among breastfeeding women in areas with a high prevalence of ZD in order to determine the need of zinc supplementation.

## 1. Introduction

Zinc is an ubiquitous mineral within the body that has catalytic, structural, and regulatory biological functions [1]. This mineral is known to be an essential micronutrient for development and normal cell activity [2]. Zinc deficiency (ZD) is one of the most common micronutrient deficiencies worldwide, affecting around 2 billion people, especially among developing countries. ZD has important consequences especially during pregnancy, lactation, and childhood development [3,4].

In spite of the physiological adjustment, which increases the zinc absorption during pregnancy and lactation, women and children are still affected by zinc deficiency due to increased nutrient requirements [5,6]. During pregnancy, ZD can affect multiple systems and could increase the risk of infections, preeclampsia, miscarriages, and adverse fetal outcomes, such as fetal growth restriction, low weight at birth, neurological malformations, and/or neurological impairment [2,4,5,6,7,8,9,10,11]. If ZD in the mothers persists after birth, particularly if their infants are exclusively breastfed, adverse infant development outcomes may occur [2,8].

Approximately 82% of mothers around the world do not have an adequate zinc intake in their diet, but the prevalence of ZD during pregnancy worldwide has not been determined [12]. It has been reported a higher prevalence of zinc deficiency among pregnant, compared to non-pregnant, women in India (65; 41%), Ethiopia (56; 34%), and Pakistan (48; 42%) [13,14,15,16]. In lactating women, the prevalence of ZD varied widely among small cohorts from different countries, such as Indonesia (25%) and Vietnam (55%) [17,18].

In Ecuador, according to the Ecuadorian National Health and Nutrition Survey (ENSANUT-ECU, 2014), 56.1% (95% CI: 54.9–57.1) of women of reproductive age have ZD. Pregnant and lactating women were not sampled in this survey [19]. On the other hand, a study conducted previously by our research group, found a prevalence of ZD of 31.4% (95% CI: 17.1–48.6) in a small cohort of mothers of children with non-syndromic cleft lip with or without cleft palate. It is important to clarify that these women did not exclusively breastfed their babies due to the oral malformation [20].

Micronutrient deficiencies have been observed in Ecuador for at least two decades, particularly in vulnerable segments of the population [21]; in part, it could be affected by globalization through multiple pathways, such as urbanization, economic growth, trade, and investments [21,22]. Processes of urbanization are associated with higher inequality and changes in the food environment, subsequently affecting the nutritional status, especially in poor people who cannot afford food of high nutritional quality [22]. Nowadays, peri-urban areas of Quito are suffering accelerated urbanization, growing in population at a higher index than the urban area [23,24].

The suggested association between maternal zinc depletion and poor outcomes of pregnancy and lactation have raised the possibility of prevention by using supplementary zinc, but results of previous studies have not been conclusive and still need to be assessed, especially focusing on populations with low dietary zinc intake [25].

The objective of the present study was to assess plasma zinc (PZn) levels and zinc supplementation rate during pregnancy, among lactating women attending to a public care center from a peri-urban Andean community of Quito, Ecuador in order to make an initial approach to the zinc status of lactating women in the region.

## 2. Materials and Methods

### 2.1. Study Setting and Participants

Between October 2015 and July 2016, a case study series was conducted in order to determine the rate of ZD among Ecuadorian mothers who have a breastfeeding infant, lived in Conocoto, and attended to the Primary Health Centre of Conocoto for regular paediatric and/or gynaecological check-ups. This health center belongs to the Ministry of Public Health which offers its services to the general population especially to people who have no private or social insurance [26].

Conocoto is one of the 33 rural parishes of Quito, the capital of Ecuador, located in the Andean region. Today, it is a heterogeneous territory with extensively urbanized areas and a few rural neighbourhoods pressed by growing urbanization. According to the last national census conducted in 2010, in Conocoto there were 82,072 inhabitants of whom 42,381 were women, with a population density of 1594 people/km^2^ and a birth rate of 16.98 [27]. Of the 33 rural parishes of Quito, eight have been characterised as peri-urbans [23].

We collected pertinent clinical information and a single blood sample to quantify zinc levels from the mothers; after they read and signed an informed consent.

Inclusion criteria were women who lived in Conocoto and had a child who was being fed mainly with breast milk. By mainly, we mean that at the time of the interview at least 90% of the children’s diet was breast milk, but three or fewer times a week some of them received other types of foods, such as “coladas” (a beverage made out of water and any kind of cereal flour), water, or formula.

As exclusion criteria, we considered the following: diagnosis of any chronic disease, any malformation in the index child, use of oral contraceptives, consumption of alcohol, cigarettes or any recreational drug at any time, any respiratory or gastrointestinal infection during the fifteen days prior to sampling and use of pharmacological therapy within this period. Additionally, we excluded pregnant women, non-fasting women, women with any disability that interferes with sample collection, and women who consumed alcohol or performed extreme exercise the night before sampling, factors that were contemplated by ENSANUT-ECU. We followed these guidelines following the methodology of a previous report of our research group [20].

This study was conducted following the Declaration of Helsinki, approved by the Bioethics Committee of the Universidad Internacional del Ecuador and registered it under the number 04-2013.

### 2.2. Measurements

Maternal variables recorded were age, educational level, number of pregnancy check-ups, and intake of vitamin and mineral supplements. In regards to vitamin and mineral supplements, we asked at what time during pregnancy they started consuming it, doses, frequency, and the form of the supplement ingested. Infant variables registered were age at the time of the interview, sex, weight at birth, length at birth, and gestational age at birth. The weight, length, and gestational age at birth were obtained from hospital records. All other information was based on self-report by the mothers.

### 2.3. Collection of Blood Samples and Determination of Zinc Levels

A single blood sample was obtained between 8 and 10 a.m. from fasting mothers using tubes coated with lithium heparin and a gel separator (BD Vacutainer^®^ PST^TM^, Becton-Dickinson Inc, Franklin Lakes, NJ, USA). The tubes were centrifuged for 10 min and refrigerated at 2–8 °C during 3–6 h. In the laboratory, the plasma was separated into additional tubes and stored at −80 °C. Plasma samples contaminated with lysed red blood cells were discarded. Then, prior to zinc quantification, the refrigerated samples were thawed at room temperature (20–24 °C), and mixed with a vortex. PZn were measured by flame-atomic-absorption spectrophotometry (AAnalyst 400, PerkinElmert, Billerica, MA, USA), the coefficient of variation for the essay was 6.2%. A standard solution of zinc in 1000 ± 4 mg/L of nitric acid (Fluka™ Analytical, Steinheim, Germany) (2% p/p) was used for the calibration curve with dilutions from 25 to 300 ppb. All chemicals were of analytical grade purity (Type I water, nitric acid 69%). Zinc quantification of all the samples was performed at the same time in dust-free fume hoods with minimal contact with air. We followed the same criteria that in our previous report [20].

Previous reports suggest 10.70 µmol/L as the cut-off point for zinc deficiency in fasting females over 10 years-old [6,28], and this value was utilized as the cut-off point by ENSANUT-ECU [19]. Therefore, we adopted this cut-off point in order to determine ZD in the present study and to compare it with the general population of Ecuadorian women of reproductive age.

### 2.4. Data Processing

The sample was evaluated as one group and divided in two subgroups based on the mothers’ declaration of zinc consumption during pregnancy. The data were analyzed using the Statistical Package for Social Science (SPSS) Statistics for Windows, version 20.0 (IBM Corp., Armonk, NY, USA). Correlations were assessed by Spearman’s rho, and *p* < 0.05 was considered as statistical significant. The *t*-test was used to compare the plasma zinc level means of the supplemented and non-supplemented groups.

In order to compare the prevalence of ZD in our sample with the reported among women of reproductive age by ENSANUT-ECU, we used the likelihood ratio and its corresponding statistic (G^2^); *p* < 0.05 was considered as statistical significant.

## 3. Results

We collected data and blood samples from 64 mothers, 3.2 (1.8) months postpartum. The mothers were between 15 and 39 years old with a mean age of 26.8 (6.4) years old. The majority of their infants were full-term (93.8%), and had adequate weight (76.6%) and length (62.5%) at birth (Table 1).

The percentages of women who consumed folic acid, iron, and zinc during pregnancy were 96.9%, 93.8%, and 31.2%, respectively (Table 1). The participants who reported ingesting zinc during pregnancy affirmed to have consumed zinc as part of a multivitamin supplement, but the majority of them were not able to accurately provide the doses and frequency of the supplements consumed and it was not possible to analyze this data.

The PZn levels were between 3.9 to 13.0 µmol/L, the mean PZn concentration was 9.3 (1.8) µmol/L. The first, second, third, and fourth quartiles were 8.1, 9.2, 10.3, and 13.0 µmol/L, respectively. We found ZD in 52 women with a prevalence of ZD of 81.2% (95% CI: 71.7–90.9), which is statically different to the reported among women of reproductive age by ENSANUT-ECU of 56.1% (95% CI: 54.9–57.1) (G^2^ = 18.23; *p* < 0.05) [19]. There was no difference between the means of plasma zinc levels of the supplemented and non-supplemented groups (*t* = 1.34; *p* > 0.05).

## 4. Discussion

The prevalence of ZD worldwide is unknown. Nevertheless, a global inadequate zinc intake rate of 17.3% has been estimated with a high geographical variation. The Andean region, which Ecuador is part of, has an estimated inadequate zinc intake of 17.0% [29]. ZD is widespread especially among developing countries affecting mainly children, and pregnant and lactating women [2,3,5,9], and it is expected to especially affect people living in areas of high urbanization that have been associated with higher levels of inequality and subsequent micronutrient deficiencies [21,22].

Many studies have found that ZD is higher among pregnant than non-pregnant women [13,14,15,16] and that zinc levels between pregnancy and postpartum periods do not differ significantly [30], which raises the need to evaluate zinc status among pregnant and lactating women in Ecuador, where high rates of ZD have been reported [19].

In Ecuador, the prevalence of ZD reported by ENSANUT-ECU among women of reproductive age from the general population is 56.1% and varies across the country, being higher in the urban coast (62.9%) and lower in the rural highlands (46.6%). In Quito, the prevalence of ZD is 55.6% [19].

Our results showed a higher prevalence of ZD among lactating women than in non-pregnant non-lactating women of reproductive age reported by ENSANUT-ECU [19], which agrees with previous reports from India, Ethiopia, and Pakistan. However, it is worth clarifying that the studies performed in Ethiopia and Pakistan evaluated zinc deficiency in both groups which were part of the same cohort [14,15,16]. Additionally, it is important to note that we are comparing two groups with different physiological status because lactating women are in a period of high metabolic demand in order to supply milk for their offspring [5,6].

Another factor that could be related to the high prevalence of ZD in our sample is the fact that nearly all the participants received iron supplements during pregnancy. The competition between iron and zinc absorption is well known, which means that PZn levels will be lower when iron is concurrently supplemented [6,31,32].

Although a mother with ZD most likely would have breast milk with a normal zinc concentration due to a physiological adjustment that tries to supply enough zinc for their offspring, it has been found that children of mothers with profound ZD are at risk of ZD and its consequences [17,31,33]. Therefore, an adequate zinc nutrition is necessary for normal pregnancy outcomes, child growth, adequate immune function and neurocognitive development [2,4,5,7,8,9,10,11]. We could not find any association between maternal zinc levels, zinc supplementation, and the anthropometry of their infants at birth, probably because our sample was small with the majority having ZD; nevertheless, the rates of ZD, premature delivery, and low weight and length at birth are not negligible [11]. Despite this, comparing the groups based on the declaration of zinc ingestion during pregnancy, we observed a slightly lower prevalence of zinc deficiency among the supplemented group than the non-supplemented group which was not statistically significant. The aforementioned statements raise the necessity to extend studies in order to assess the need of zinc supplementation among lactating women in disadvantaged groups with similar conditions.

Zinc supplementation has proven to reduce the rate of premature delivery, and improve linear growth and weight gain, especially in children of short stature, and decrease morbidity, duration, and severity of diarrhea and acute lower respiratory infection [1,8,34]. Supplementing zinc during pregnancy and lactation increases the mothers’ PZn concentration by 3% and 1%, respectively, which could protect the mothers’ nutritional status and, therefore, their children during these periods of life [35].

In spite of the aforementioned health zinc benefits, the World Health Organization (WHO) recommends zinc supplementation for pregnant women only in the context of rigorous research due to the moderate to low certainty evidence related to maternal, fetal, and neonatal outcomes, and no guidelines for zinc supplementation during lactation exist [7,36]. Nevertheless, we found that 31.2% of the participants were supplemented with zinc during pregnancy and suspended after delivery. Maternal zinc supplementation during lactation has yielded inconsistent results regarding the improvement of breast milk zinc concentrations or to demonstrate benefits for mothers and their infants [37,38]; however, considering the high rates of ZD among lactating women, it is worth extending studies to address the need of zinc supplementation identifying a profile of mothers that could benefit from this.

It is important to mention that in a previous report following the same methodology, we evaluated PZn levels in a cohort of 35 women who had a child between 1 and 12-months-old with an oral malformation, reporting a prevalence of ZD of 31.4; these results were probably due to the use of formula to feed the children because the oral malformation causes breastfeeding difficulties [20]. Lactation is a risk factor for zinc deficiency and the recommended dietary allowance is higher among pregnant and lactating women than in non-pregnant non-lactating women [6]. Therefore, the use of formula could have protected the mothers of children with the oral malformation from developing ZD in the postpartum period and could explain its lower ZD prevalence compared with the women of the present study.

Our study had several limitations. First, the sample corresponds to a small group of women from a single peri-urban community of Quito which does not allow extending these results, as the prevalence of zinc deficiency could differ by taking into account differences in socio-economic status or geographical location, however, this could be an example of what is happening among women from communities under pressure of accelerated urbanization where inequality levels are high [21,22].

Second, even though women who were going through any type of clinical infection or disease were excluded from the study, we did not assess any inflammatory marker, such as alpha 1 glycoprotein, C-reactive protein and interleukin-6; that would allow exclusion of women with an inflammatory process that could be related to decreased PZn [39,40]. It is also important to note that postpartum women, in general, have a more active immune system and higher serum levels of proinflammatory cytokines, and it is somewhat more active among breastfeeding than formula-feeding women [41]. Third, we were unable to register the doses, frequency, and forms of zinc ingested as, most of time, it was part of a multivitamin and mineral supplement; this did not allow for a deep analysis regarding zinc supplementation. Finally, we included only healthy women, therefore, this prevalence would likely increase if women with any comorbidity were included.

The results of this study raise the necessity to evaluate zinc supplementation during pregnancy and the extension to the lactation period in breastfeeding women who attend public care centers for their controls after delivery living in communities experiencing accelerated urbanization, such as Conocoto. Furthermore, we recommend extending studies in the Andean region and in areas with a suspected or confirmed high prevalence of ZD to document levels of zinc among breastfeeding women.

## Figures and Tables

**Table 1:** Plasma zinc levels, maternal and infant characteristics of zinc supplemented and non-supplemented women during their pregnancies (*n* = 64).

Recorded Variables	All the Sample *n* = 64	Women Who Did Not Received Zinc Supplements *n* = 44	Women Who Received Zinc Supplements *n* = 20	ρ (Rho) de Spearman	*p*−Value
**Maternal variables**					
Age (years, mean ± SD) *	26.8 ± 6.5	26.1 ± 6.8	28.4 ± 5.7	0.17	0.17
Level of education (%)					
Elementary school only	21.9	22.7	20.0	0.03	0.81
High school or any superior level	78.1	77.3	80.0		
Number of prenatal check−ups (%)					
<5	6.2	6.8	5.0	0.04	0.79
≥5	93.8	93.2	95.0		
Consumption of iron during pregnancy (%)					
Yes	93.8	90.9	100	0.17	0.17
No	6.2	9.1	0		
Consumption of folic acid during pregnancy (%)					
Yes	96.9	95.5	100	0.12	0.34
No	3.1	4.5	0		
PZn (µmol/L, mean ± SD) *	9.3 ± 1.8	9.07 ± 1.7	9.72 ± 2.0		
Zinc deficiency *n* (%)	52 (81.2)	37 (84.1)	15 (75)	−0.11	0.40
**Infant variables**					
Infants’ age (months, mean ± SD) *	3.2 ± 1.8	3.3 ± 1.8	3.0 ± 1.7		
Sex (Male/Female)	32/32	22/22	10/10		
Gestational age at birth (%)					
37 weeks or more	92.2	90.9	95.0	0.07	0.58
Less than 37 weeks	7.8	9.1	5.0		
Weight at birth					
2500 g or more	78.1	79.5	75.0	−0.05	0.69
Less than 2500 g	21.9	20.5	25.0		
Length at birth (%)					
48 cm or more	62.5	61.4	65.0	0.04	0.79
Less than 48 cm	37.5	38.6	35.0		

* *t* was calculated with non-statistical significant results. SD: Standard deviation.
